# Periaortitis and Retroperitoneal Fibrosis in a Patient With a Normal IgG4 Level: A Case Report and Review of the Literature

**DOI:** 10.7759/cureus.114453

**Published:** 2026-08-13

**Authors:** Khaled Mohamed S Alarbi, Afra Mohamed, Mohammed Almubarak S Elkhidir, Esraa M Ata, Roba K Younis, Hana Nishan, Sushil Niraula, Jaseem Sirajudeen, Nishan K Purayil, Vamanjore A Naushad

**Affiliations:** 1 Department of Medicine, Hamad Medical Corporation, Doha, QAT; 2 Department of Clinical Medicine, Weill Cornell Medicine Qatar, Al Rayyan, QAT; 3 Department of Internal Medicine, Hamad General Hospital, Doha, QAT; 4 Department of Clinical Medicine, College of Medicine-QU Health, Qatar University, Doha, QAT

**Keywords:** abdominal pain, fibrosis, igg4-related disease, periaortitis, retroperitoneal fibrosis

## Abstract

We present a case of IgG4-related periaortitis with retroperitoneal fibrosis (RPF) in a 66-year-old man with a complex medical history, including coronary artery disease, hypertension, and prediabetes. The patient presented with abdominal and flank pain, and imaging revealed inflammatory changes surrounding the abdominal aorta and splenic artery. Treatment with corticosteroids resulted in significant symptomatic improvement. This case is compared with previously reported cases of periaortitis, including those associated with endovascular aortic repair (EVAR) and idiopathic IgG4-related disease (IgG4-RD). We highlight the similarities and differences in clinical presentation, underlying etiology, and response to treatment. In this case, the diagnosis was based on the clinical presentation, radiological findings, and favorable response to corticosteroid therapy, despite normal serum IgG4 levels. This case highlights the importance of considering IgG4-RD in the differential diagnosis of periaortitis and RPF, even when laboratory findings, including serum IgG4 levels, are within the normal range.

## Introduction

Retroperitoneal fibrosis (RPF) is a rare condition characterized by the proliferation of fibrous tissue within the retroperitoneal space, particularly around the infrarenal portion of the aorta and the iliac arteries, commonly resulting in the encasement of the ureters or other abdominal organs [1]. RPF may be primary (idiopathic) or secondary. Idiopathic RPF, which is more common than secondary RPF, accounts for approximately 70% of cases and may be either IgG4-related or non-IgG4-related. Secondary RPF may occur as a result of infection, malignancy, medications, or retroperitoneal hemorrhage.

The incidence of idiopathic RPF is estimated at 0.1-1.3 per 100,000 persons per year [2,3]. It occurs most commonly in individuals between 40 and 60 years of age and is more prevalent in males [3,4]. Inflammatory abdominal aortic aneurysm (IAAA), a manifestation of chronic periaortitis (CP), accounts for approximately 3%-10% of all abdominal aortic aneurysms [5]. Idiopathic RPF is considered part of the disease spectrum of CP, which is characterized by fibrosis and inflammation surrounding the aorta and iliac arteries [1].

CP is a fibroinflammatory condition characterized by periaortic and peri-iliac inflammatory tissue that frequently entraps retroperitoneal structures, particularly the ureters, resulting in ureteral obstruction and potentially renal failure. CP encompasses both non-aneurysmal forms, such as idiopathic retroperitoneal fibrosis (IRF), and aneurysmal forms, including IAAAs and peri-aneurysmal retroperitoneal fibrosis (PRF) [1-3]. It may occur as an isolated condition or in association with systemic immune-mediated diseases, such as small-vessel vasculitis, systemic lupus erythematosus, and rheumatoid arthritis. It is also frequently associated with organ-specific autoimmune diseases, including Hashimoto's thyroiditis [1,4-6]. In idiopathic RPF, the disease typically follows a slowly progressive course, with clinical manifestations primarily resulting from ureteral compression. Common symptoms include nonspecific abdominal pain and renal impairment [6-8]. Corticosteroid therapy is generally considered the first-line treatment for idiopathic RPF. Other therapeutic options include nonsteroidal anti-inflammatory drugs, tamoxifen, antifibrotic agents such as colchicine, immunosuppressive therapy, and radiotherapy. Surgical intervention may also be required in selected cases [6-8]. CP may occur as a manifestation of IgG4-related disease (IgG4-RD), a systemic fibroinflammatory condition that can involve the retroperitoneum and large vessels. Here, we present a case of IgG4-related periaortitis in which serum IgG4 levels were within the normal range but the patient demonstrated a favorable clinical response to corticosteroid therapy.

## Case presentation

A 66-year-old man with a 40-pack-year smoking history was admitted with vague abdominal and left flank pain, rated 6/10 on the pain scale, accompanied by projectile vomiting. The abdominal pain was predominantly periumbilical and non-radiating, whereas the left flank pain radiated to the groin. He denied urinary symptoms, including dysuria, increased frequency, urgency, or changes in urine color. However, he reported a reduction in urine output compared with his usual baseline. He denied recent weight loss, fever, or other systemic symptoms but reported a history of chronic constipation.

His medical history included hypertension, dyslipidemia, coronary artery disease, and prediabetes. He had previously undergone multiple coronary angiograms and percutaneous coronary interventions.

On general physical examination, his vital signs were within normal limits. Abdominal examination revealed marked tenderness over the left flank and central abdomen without guarding. Bowel sounds were normal. Examination of the other systems was unremarkable.

Diagnostic assessment

Initial laboratory investigations revealed a normal white blood cell count of 6.1 × 10³/µL (reference range: 4,500-11,000 cells/µL) and platelet count of 179 × 10³/µL (reference range: 150,000-450,000 cells/µL), but showed mild anemia, with a hemoglobin level of 11.5 g/dL (reference range: 13.8-17.2 g/dL for males). The C-reactive protein (CRP) level was 3.4 mg/L (reference range: <5 mg/L), while the D-dimer level was elevated at 0.83 µg/mL (reference range: <0.5 µg/mL). The serum IgG level was 12.21 g/L (reference range: 7-16 g/L). Detailed laboratory parameters are presented in Table 1.

**Table 1:** Lab results on day of admission and on follow-up Ig: immunoglobulin. Dashes (-) indicate that the test was not done.

Laboratory test	Patient results			Reference range
	On admission	On day 24	On one-month follow-up	
White blood cells (WBC)	6.1	11	13	4.0-10.0 × 10^3^/µL
Hemoglobin	11.5	15.8	15.2	12-15 mg/dL
Platelets	179	221	200	150-410 × 10^3^/µL
C-reactive protein (CRP)	3.4	2	2	0.0-5.0 mg/L
Erythrocyte sedimentation rate (ESR)	16	10	2	2-37 mm/hr
Creatine kinase (CK)	47	-	-	39-308 U/L
Myoglobin	33	-	-	28-72 ng/mL
Creatinine	69	-	-	62-106 umol/L
Alanine aminotransferase (ALT)	14	17	12	0-41 U/L
Aspartate aminotransferase (AST)	20	17	11	0-40 U/L
Alkaline phosphatase	63	59	60	40-129 gm/L
Total bilirubin	4	7	8	0-21 umol/L
Albumin	36	38	43	35-50 gm/L
Total IgE	180	-	-	0-114 kU/L
IgG	12.21	-	-	7-16 gm/L
IgG sub 1	6,214	4,151	4,279	3,824-9,286 mg/L
IgG sub 2	4,013	3,367	2,648	2,418-7,003 mg/L
IgG sub 3	1,152	497	773	218-1,761 mg/L
IgG sub 4	390	203	75	39-864 mg/L
C3	1.38	-	-	0.9-1.8 gm/L
C4	0.22	-	-	0.1-0.4 gm/L
Serum protein electrophoresis	Unremarkable	-	-	

**Figure 1:**
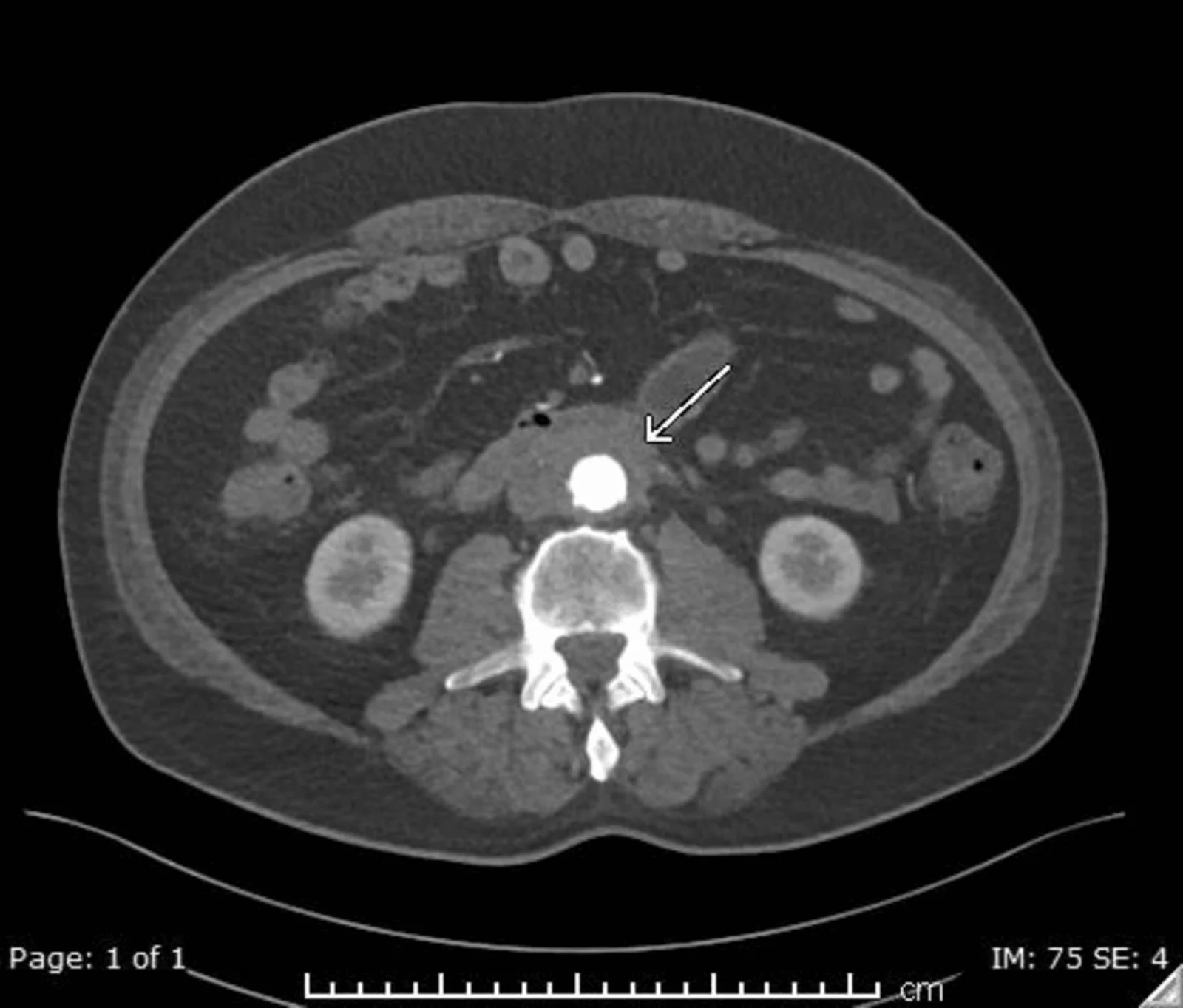
CT angiography of the abdomen (coronal plane) showing periaortic inflammation (arrow)

**Figure 2:**
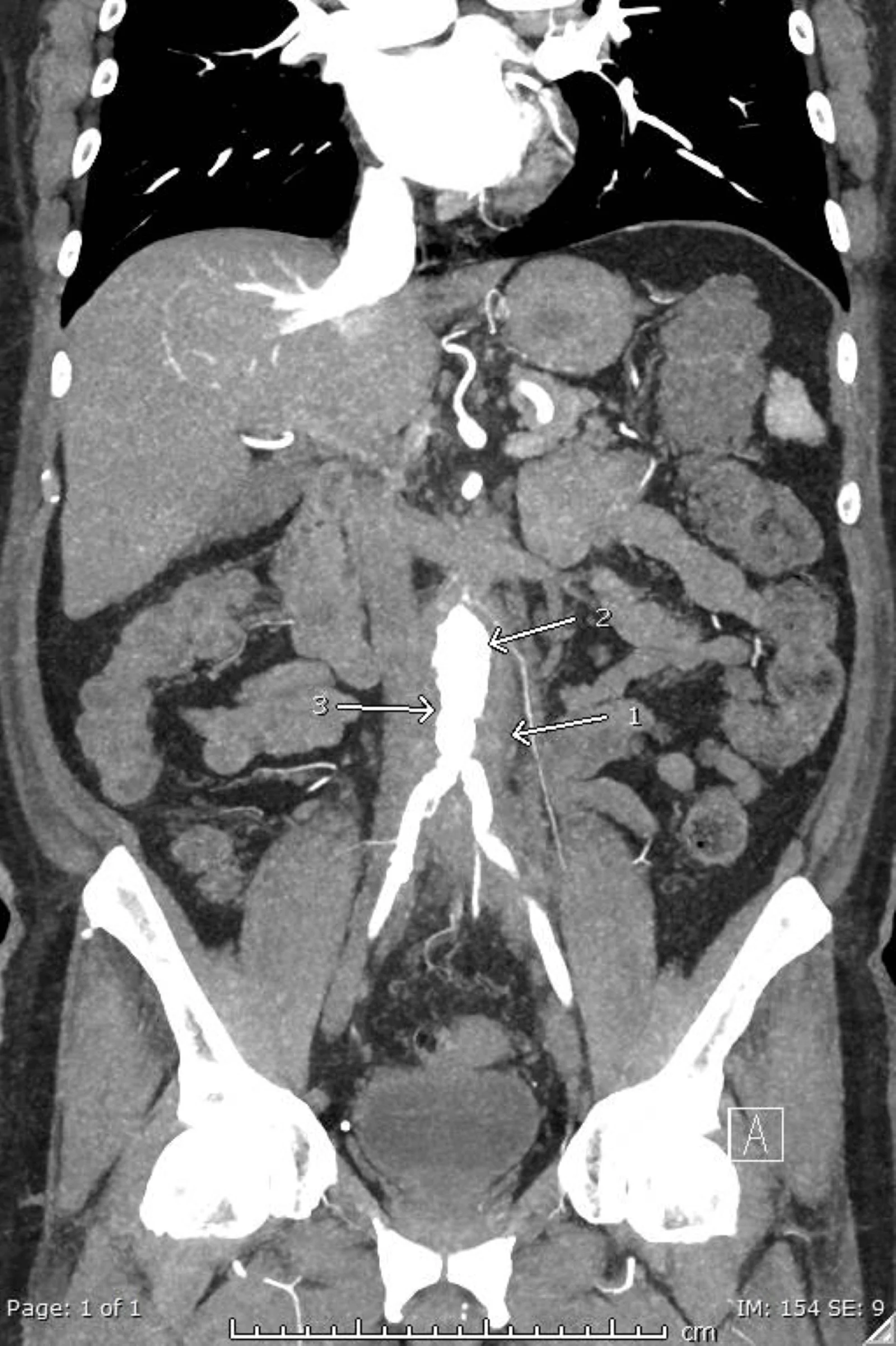
CT angiography of the abdomen (sagittal plane) showing periaortic inflammation and retroperitoneal fibrosis (arrows)

A computed tomography (CT) scan of the kidneys, ureters, and bladder showed no urinary calculi. Subsequent CT angiography of the abdomen and pelvis revealed aortic mural thickening extending inferiorly from the level of the renal arteries, with areas of luminal narrowing and mild dilatation. Mildly enhancing soft-tissue density was also noted in the periaortic and retroperitoneal regions at the infrarenal level, suggestive of CP with associated retroperitoneal fibrosis (Figures 1, 2).

**Figure 3:**
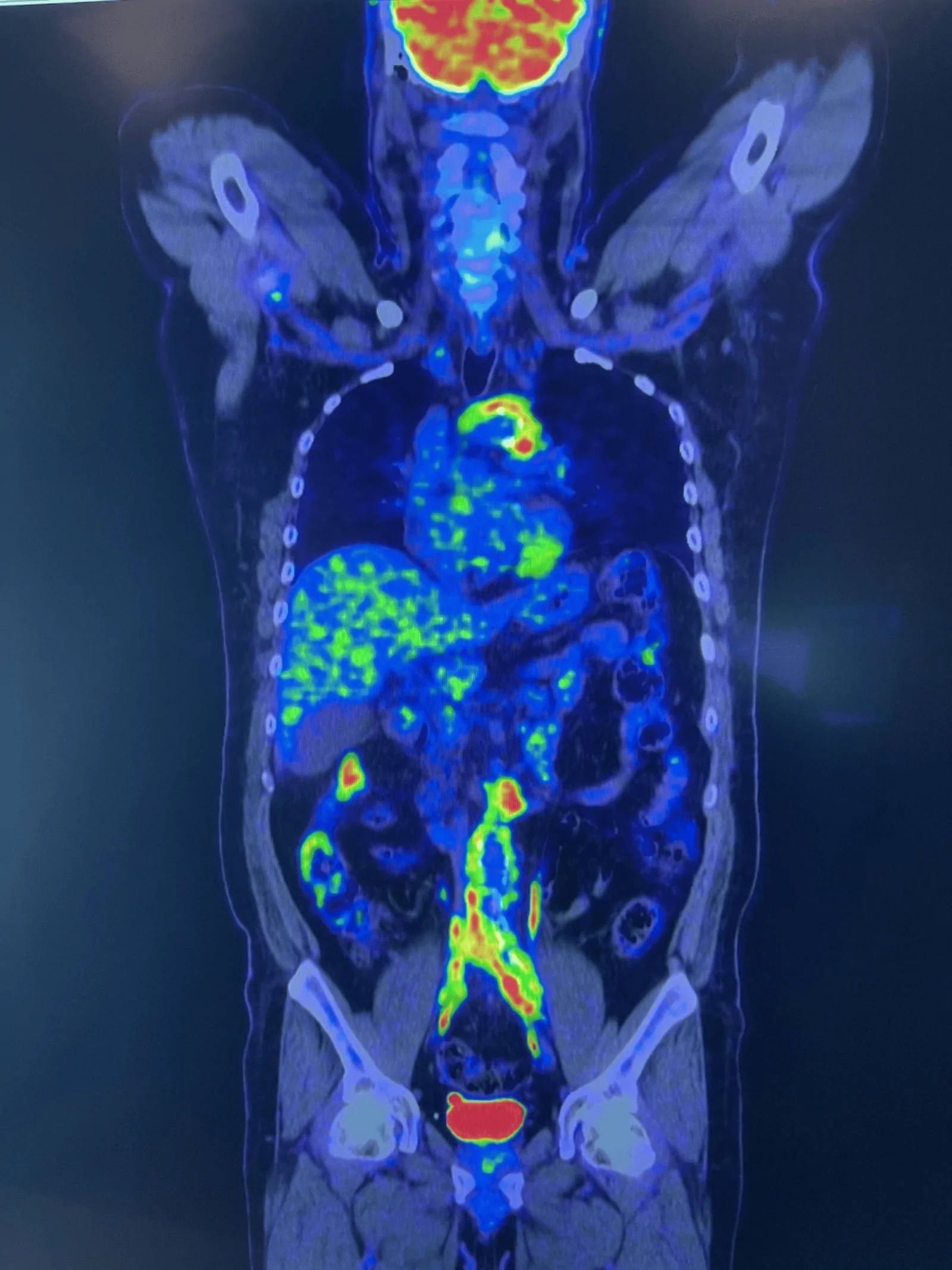
PET-CT scan (coronal plane) showing intense inhomogeneous FDG uptake in the aortic arch wall and intense homogeneous FDG uptake in the distal abdominal aortic wall and surrounding soft-tissue sleeve going below bifurcation level along the iliac arteries PET-CT: positron emission tomography-computed tomography, FDG: fluorodeoxyglucose.

**Figure 4:**
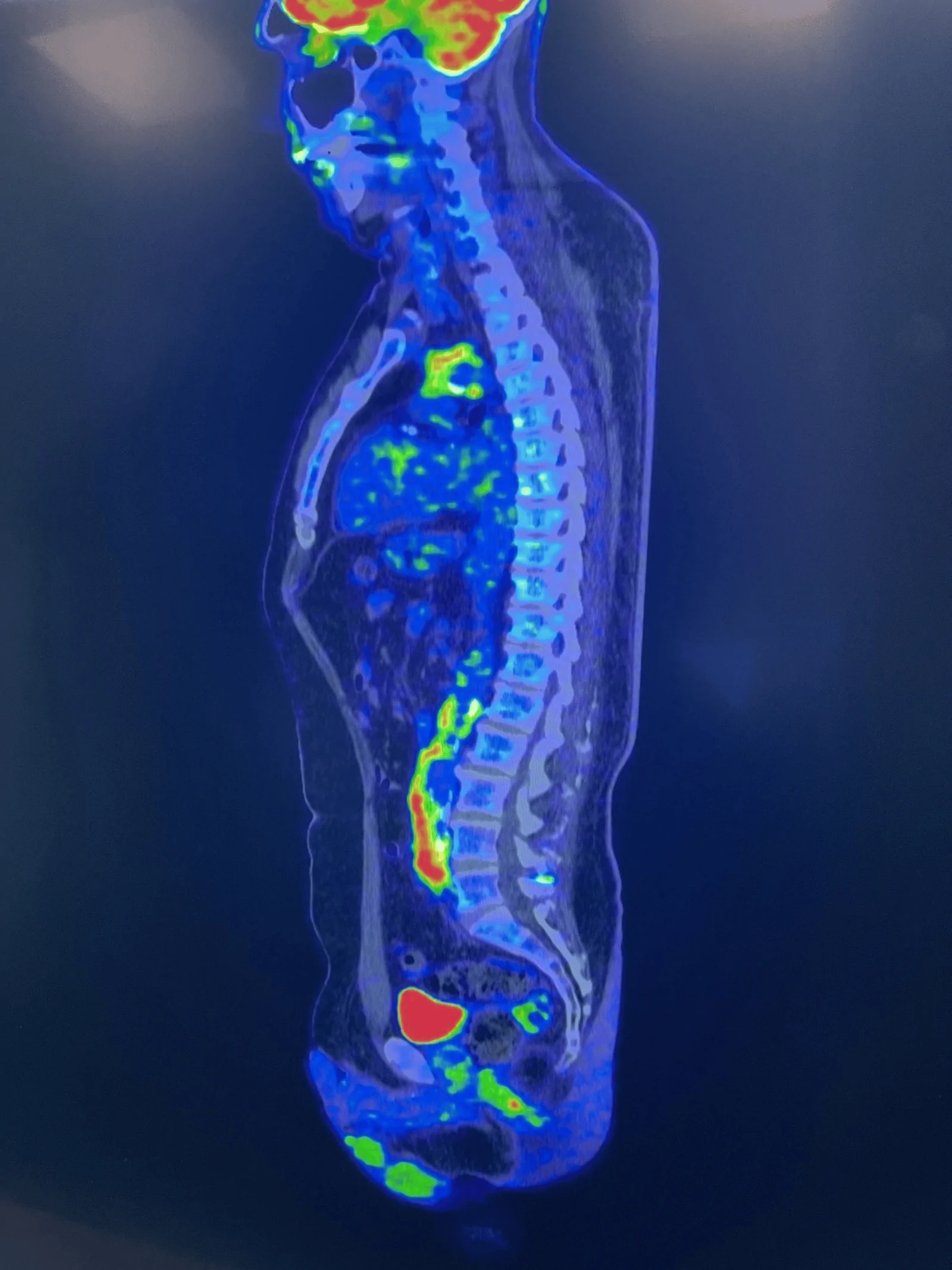
PET-CT scan (sagittal plane) showing intense inhomogeneous FDG uptake in the aortic arch wall and intense homogeneous FDG uptake in the distal abdominal aortic wall and surrounding soft-tissue sleeve going below bifurcation level along the iliac arteries

A positron emission tomography-computed tomography (PET-CT) scan demonstrated intense, inhomogeneous fluorodeoxyglucose (FDG) uptake along a 12-cm segment of the aortic arch wall. There was also intense, inhomogeneous FDG uptake in the distal abdominal aortic wall, with a surrounding soft-tissue sleeve extending below the aortic bifurcation along the iliac arteries for a total length of more than 20 cm. In addition, there was pathological FDG uptake in the wall of the splenic artery. No peripheral lesions amenable to biopsy were identified. The PET-CT findings supported an active inflammatory process involving the aortic wall at the aortic arch and distal abdominal aorta (Figures 3, 4).

Although the serum IgG4 level was within the normal range, considering the severity of the patient’s symptoms, the characteristic findings on CT angiography of the abdomen and PET-CT, and the absence of peripheral lesions suitable for biopsy, a diagnosis of IgG4-related periaortitis was made following consultation with a rheumatologist. The patient was commenced on oral prednisolone at a dose of 50 mg daily (1 mg/kg/day) and azathioprine 50 mg daily. Five days after initiating treatment, the patient reported a 70%-80% improvement in pain, indicating a favorable therapeutic response. This clinical improvement is consistent with the typical response observed in IgG4-RD following glucocorticoid therapy. The patient was discharged after an eight-day hospital stay. At discharge, he was advised to gradually taper the prednisolone dose and increase the azathioprine dose by 50 mg every two weeks, up to a maximum of 150 mg daily.

At the three-week outpatient follow-up, the patient was asymptomatic and clinically well. A repeat PET-CT scan performed seven months later demonstrated regression of FDG uptake in the aortic arch, with partial regression of FDG uptake in the aortic wall and surrounding soft tissues. Complete resolution of FDG uptake in the splenic artery was also observed. Prednisolone was discontinued seven months after discharge, while azathioprine was continued at a dose of 150 mg daily. The complete resolution of symptoms following treatment, together with the improvement in the findings on repeat PET-CT, further supported the diagnosis of IgG4-related periaortitis in this patient.

## Discussion

In-depth review of literature

Idiopathic or primary RPF is an immune-mediated disease that accounts for approximately 70% of cases. It may occur in isolation or in association with other autoimmune or inflammatory conditions, such as fibroinflammatory IgG4-RD [9,10]. CP encompasses periaortic RPF, in which the aortic caliber is normal, and IAAA, in which the aorta is dilated. Because of the similarities in their clinical characteristics and histological findings, these conditions are generally considered part of the same disease spectrum [11,12]. Most cases of idiopathic RPF are classified as CP because of their typical localization in the periaortic and iliac regions. However, idiopathic RPF with atypical localizations, such as the pelvis or peripancreatic region, cannot be classified as CP.

Etiology and pathophysiology

The etiology of RPF remains largely unknown [13,14]. Autoimmune diseases, chronic inflammatory disorders, infections, medications, and malignancies have been implicated; however, no specific etiology has been unequivocally established [15,16]. Therefore, the term idiopathic RPF remains appropriate, reflecting the limited understanding of its underlying pathogenesis [15,17]. Cadaveric studies of patients with acute RPF demonstrated that the disease process involved not only the retroperitoneal fibrous tissues but also the abdominal aorta, suggesting that the disease may involve the segment of the infrarenal aorta that traverses the retroperitoneal space. The development of aneurysms in the affected portion of the aorta further suggested a potential relationship between the connective tissue surrounding the aorta and inflammation of the aortic wall [18,19].

Recent research has demonstrated an association between idiopathic RPF and IgG4-RD, a distinct group of disorders characterized by infiltration of IgG4-positive plasma cells and fibrosis involving the retroperitoneal tissues [20]. IgG4-RD encompasses several conditions that were previously considered separate pathological entities, highlighting the clinical and pathological similarities among these disorders. Consistent with the characteristic features of IgG4-RD, the mononuclear inflammatory cell infiltrate and fibrosis observed in affected tissues share similar histological features across different organs. The fibrosis associated with RPF and that observed in IgG4-RD aortitis may show overlapping histological features and may coexist in the same patient [20,21].

Autoimmune Factors

Different circulating cytokine levels have been investigated in patients with periaortitis and RPF. In general, these patients have been reported to have elevated levels of interleukins 2, 4, and 6, with normal levels of tumor necrosis factor-alpha. Neopterin levels have also been investigated as a potential marker of inflammatory disease [22]. Neopterin is secreted primarily by human monocytes and macrophages and has been used as a marker in the assessment of infections and other inflammatory conditions [23]. However, a small study investigating neopterin levels in patients with periaortitis or RPF did not yield conclusive results [22]. Given the inflammatory nature of these conditions, various anti-inflammatory treatment strategies have been investigated, including corticosteroids, antirheumatic agents, and surgical intervention. Although the available evidence remains inconclusive, these therapeutic approaches, particularly for secondary forms of periaortitis and RPF, require further evaluation in large prospective studies to establish their efficacy [15,22].

Infectious Causes

No infectious agent has been established as a strong candidate for the sole cause of idiopathic RPF or IgG4-RD [20,24]. Previous studies have reported the detection of infectious agents, including *Mycobacterium tuberculosis*, *Actinomyces* species, *Histoplasma* species, and viruses such as Epstein-Barr virus (EBV), hepatitis C virus (HCV), and human immunodeficiency virus (HIV), using both in situ hybridization of RPF lesions and polymerase chain reaction (PCR) analysis of circulating mononuclear cells from patients with RPF. For several years, researchers investigated the presence of EBV in RPF tissues and its potential association with other infectious agents and immune responses to these infections. However, the small number of reported cases and the lack of consensus have limited the strength of the evidence supporting this hypothesis [24]. Some studies have suggested an association between seropositivity to certain infectious agents and RPF; however, similar findings were not observed in a Japanese cohort of patients with RPF [21]. Given the absence of strong evidence supporting an infectious etiology or a clearly defined immunopathogenic mechanism, the potential role of molecular mimicry remains poorly understood [20,24].

Several groups of researchers subsequently re-examined the potential association between viral infection and IgG4-RD in small and heterogeneous cohorts of Japanese patients. In one study, a highly sensitive assay was used to detect a viral nuclear antigen, but no evidence of the antigen was identified in the paracortical lymphadenopathy specimens from Japanese patients. Studies involving patients from other East Asian populations have likewise failed to demonstrate convincing evidence of such an association. Thus, the available evidence remains insufficient to support a causal relationship between viral infection and IgG4-RD, including the hypothesis that certain viruses may induce polyclonal IgG4 elevation in related conditions. Given the widespread seroconversion associated with viral infections across different populations, the role of viral infection as a significant cause of IgG4-RD remains uncertain [21].

Clinical presentation

Periaortitis is often an incidental finding, as the majority of patients are asymptomatic at the time of diagnosis. The condition is frequently identified during imaging studies, most commonly performed before aortic aneurysm repair or abdominal surgery unrelated to the aorta [19,25,26]. When symptomatic, patients commonly present with nonspecific constitutional symptoms related to inflammation, including malaise, fever, fatigue, weight loss, anorexia, and myalgia [19,27]. Symptomatic periaortitis may predispose patients to an increased risk of vascular complications, particularly involving the aorta. Embolic events may occur as a result of plaque erosion into the aortic lumen. Back pain has been reported in up to 20% of patients and may result from adjacent inflammation involving the aortic adventitia, with subsequent radiation of pain, or from occlusion of neighboring arteries [19,27,28].

Periaortitis may also present with systemic inflammatory features, including an elevated white blood cell count, erythrocyte sedimentation rate (ESR), and C-reactive protein (CRP) level, accompanied by symptoms such as fever, night sweats, and weight loss in the absence of an identifiable infectious source [19,22,28]. In patients with idiopathic RPF, a concomitant periaortic inflammatory process may be present in up to 30% of cases. Given the potential space occupied by these fibrotic plaques within the retroperitoneum, ureteral involvement may occur, resulting in ureteral or ureteropelvic junction obstruction. A characteristic radiological appearance, sometimes referred to as the “pseudo-T collar” sign, has also been described [9,13].

Symptoms and Signs

The clinical manifestations of RPF are thought to result from the mass effect and inflammatory activity of the fibrotic process [13,29,30]. In most patients, pain is the earliest and most common symptom. Hydronephrosis and renal impairment may develop as a result of ureteral encasement [9,13]. Patients may also present with symptoms caused by venous obstruction, retroperitoneal inflammation, the development of a pelvic mass, intestinal obstruction, or vascular fistulization [13,29]. Submandibular lymphadenopathy has been reported as the most frequent extraperitoneal manifestation in patients with idiopathic, drug-related, or malignancy-associated RPF [13,31]. The association between RPF and an acute-phase inflammatory response has been well established [13,22]. Both the intensity of the inflammatory response and the number of associated symptoms appear to correlate with the extent of inflammatory activity. An association with IgG4-related disease has also been reported and may be associated with a favorable response to corticosteroid therapy [13,28].

More than 15% of patients with RPF are asymptomatic at presentation, and symptoms may not develop until complications become clinically significant or potentially life-threatening. Ureteral encasement may be clinically silent, particularly in the early stages, and the resulting hydronephrosis may initially lack specific clinical features. Persistent ureteral encasement may remain asymptomatic when the associated retroperitoneal fibrosis is limited, as the fibrosis may exert pressure on the ureter without causing complete obstruction [13].

Diagnostic imaging

Imaging plays an important role in the evaluation of periaortitis and RPF, with CT being particularly useful for characterizing the extent and distribution of disease. A relatively high frequency of periaortic lesions with a characteristic “signet-ring” appearance has been described in association with this condition. However, a history of malignancy should be carefully considered, as metastatic lymphadenopathy and primary or secondary malignant lymphadenopathy may demonstrate similar imaging features and can mimic periaortitis [25,31]. In some patients, these imaging findings may lead to diagnostic intra-abdominal surgery to exclude an underlying neoplastic process, particularly when bowel involvement is suspected. In the absence of an identifiable underlying cause, the condition may subsequently be classified as idiopathic. Calcification may be observed in extensive disease and is thought to represent a chronic or dystrophic reactive component. The absence of an aortic aneurysm or other significant aortic abnormality may also make the diagnosis more challenging and may require recognition of the characteristic periaortic imaging pattern [30,32].

Periaortitis may be suspected when imaging demonstrates a relatively bulky, sclerotic periaortic soft-tissue lesion surrounding the aorta. The distribution of the lesion may be asymmetric, partly because of the anatomical positioning of adjacent retroperitoneal structures, and may result in displacement of surrounding organs. Fibrotic tissue may cause retraction of the renal fascia and adherence of the kidney to the anterior abdominal wall. These changes can produce an abnormal appearance in the periaortic and retroperitoneal regions and may occasionally mimic a solid parenchymal mass or enlarged lymph node. These imaging characteristics have led to the description of the “signet-ring” appearance as a potential imaging feature that may help distinguish idiopathic or inflammatory retroperitoneal fibrosis from neoplastic lymphadenopathy. Similar perivascular soft-tissue masses can occur around the major vessels in conditions such as lymphoma and metastatic disease; therefore, these malignant causes should be considered in the differential diagnosis [22,33].

Management

Therapeutic options for periaortitis include surgical intervention, immunosuppressive agents, corticosteroids, mesalamine, and observation [34,35]. Proximal extraluminal dilatation identified on cross-sectional imaging performed to assess strictures is an important factor in determining the need for surgical treatment [36]. Strictures involving the proximal duodenum or ureters, as well as compression of major arteries or intra-abdominal organs, are important factors that may influence clinical outcomes [35,37]. The increasing recognition of inflammatory mechanisms, the need to limit the duration and cumulative exposure to immunosuppressive agents, and the potential anti-inflammatory and antifibrotic effects of therapies targeting macrophage activity may explain the growing number of therapeutic agents being investigated for the treatment of periaortitis [38,39].

The decision to initiate corticosteroids or other immunosuppressive agents should be based primarily on the severity of symptoms and the extent of disease activity [34,35]. In most cases, particularly those with a relatively benign natural history, patient discomfort can be managed through dose adjustment and, when appropriate, modification of supportive therapy [35,40]. Patients with chronic, recurrent, or severe obstructive complications, a severe inflammatory response, clinically significant active disease despite being asymptomatic, thoracic involvement, or extra-abdominal or vascular involvement that threatens organ function or quality of life may benefit from immunosuppressive therapy. Steroid-sparing agents may be considered in patients who have contraindications to corticosteroids or in those in whom prolonged corticosteroid therapy is undesirable [34,38,40]. Steroid-sparing therapy may be used for 6-12 months in patients with the potential for spontaneous reduction in disease activity, or earlier in those with significant obstruction or progressive disease [34,38,40]. This may be followed by gradual dose reduction over 3-6 months. Once symptoms are controlled, corticosteroid tapering should be individualized and may continue for approximately 6-18 months, depending on the clinical response and disease activity [35]. Disease recurrence may occur following reduction or discontinuation of therapy; however, many patients remain clinically stable during follow-up [34,40]. For patients experiencing frequent relapses, additional immunosuppressive or steroid-sparing agents may be considered [34,36]. In selected patients, corticosteroids may be withheld in favor of close clinical and radiological monitoring, particularly when the patient is asymptomatic and has no evidence of active disease or significant obstruction [36,41]. In cases of obstruction, inadequate clinical response to high-dose corticosteroid therapy, or inability to taper corticosteroids within six months, a trial of an alternative immunosuppressive agent may be considered [35,42]. Clinical symptoms and radiological assessment of obstruction are important endpoints for monitoring disease activity and response to treatment in fibrotic disorders [35,37].

Medical Treatment

Glucocorticoids constitute the mainstay of treatment for patients with idiopathic RPF and periaortitis [34,39]. In patients presenting with newly diagnosed or unstable disease and severe symptoms, high-dose corticosteroid therapy should be initiated promptly, followed by gradual dose tapering [34,35,38]. The duration of corticosteroid therapy required to achieve symptom control varies considerably, although treatment is generally continued for approximately 3-6 months [35]. The dose and duration of corticosteroid therapy in patients with relapsing disease should be individualized according to the severity of clinical manifestations and disease activity [34]. Once clinical stability has been achieved, corticosteroids can be gradually tapered according to an individualized or predefined regimen. The optimal corticosteroid tapering schedule remains a matter of debate [35].

For patients receiving a mean prednisone dose of 20 mg/day for at least three months, one suggested tapering regimen is to reduce the prednisone dose by 2.5 mg every two weeks until a dose of 7.5 mg/day is reached, followed by reductions of 1.25 mg every two weeks until complete discontinuation [35].

In patients who are not candidates for long-term corticosteroid therapy because of contraindications, glucocorticoid intolerance, or an inadequate response to glucocorticoids, second-line immunosuppressive therapy may be required [38,42]. Immunomodulatory or immunosuppressive agents that have demonstrated activity in RPF include azathioprine, tacrolimus, methotrexate, mycophenolate mofetil (MMF), cyclophosphamide, and interleukin-1 receptor antagonists [35,38,42]. Several case reports and a small number of case series have demonstrated favorable responses to MMF in patients with corticosteroid-dependent or relapsing disease; however, clinical evidence remains limited, and further clinical studies are needed [35,38]. Furthermore, patients with newly diagnosed or unstable disease that is refractory to corticosteroids may benefit from the addition of methotrexate [38,42].

Conventional disease-modifying antirheumatic drugs, such as azathioprine, or more potent immunosuppressive agents, such as ciclosporin or cyclophosphamide, may be considered when patients have contraindications to, intolerance of, or an inadequate response to methotrexate [35,38]. The response to azathioprine is highly variable but may occur relatively rapidly in some patients [38]. Other disease-modifying therapies, including anti-tumor necrosis factor (TNF) biological agents, have also been investigated [38,43]. In patients with relapsing disease who cannot tolerate or should not receive further glucocorticoid therapy, tacrolimus may be considered as a steroid-sparing and disease-modifying agent [38,39]. However, some patients with idiopathic RPF remain refractory to immunosuppressive therapy with azathioprine, tacrolimus, or methotrexate [38].

Surgical Interventions

Generally, the rate of surgical intervention among patients with primary RPF is relatively low. Indications for surgical intervention include urinary tract obstruction or hydronephrosis, progression of hydronephrosis during follow-up, impaired renal function secondary to obstruction, coeliac artery or mesenteric ischemia, and suspected inflammatory or proinflammatory abdominal processes [34,37]. The efficacy of surgery in patients with primary periaortitis remains uncertain. However, there is evidence suggesting potential benefits from surgical intervention during the acute phase of the disease, particularly when there is occlusion of critical vessels such as the superior mesenteric artery [34].

In patients with aortitis, aneurysms may develop in any segment of the aorta. Involvement of the abdominal aorta may also result in compression of the inferior vena cava [44]. Management of these complications may require surgical intervention, which can be technically challenging because of granulomatous changes, aortoiliac shortening, and involvement of the distal anastomotic site [37]. The presence of retroperitoneal fibrosis can make surgical dissection difficult and may reduce the effectiveness of the intervention [45]. In addition, visceral vessel involvement may be present during the preoperative period [37]. Abdominal pain and a temperature exceeding 37.5 °C have been reported as predictors of mortality during the early postoperative period. CT findings, abdominal ultrasonography, a CRP level of 100 mg/L, and anemia have also been associated with early re-occlusion [45].

Prognosis, complications, and limitations

Prognosis

Due to the often asymptomatic nature of the disease in its early stages, prompt diagnosis and treatment are essential to achieve symptom resolution and prevent progression to complications. Furthermore, IgG4-RD is a fibroinflammatory condition that generally responds well to treatment during the early inflammatory stages. In contrast, once established fibrosis develops, the response to treatment may be limited, potentially resulting in persistent disease and an increased risk of complications.

Complications

Despite the relatively high incidence of chronic aortic dissection, only a few cases of IgG4-related periaortitis have been reported. In these cases, aortic dissection typically developed either immediately or within a few days of symptom onset. Despite timely surgical intervention, some patients died as a result of catastrophic complications [36]. This may be attributable to rapid local disease progression, leading to compression of vital structures [23,37], or to venous thrombosis involving the inferior vena cava, resulting in vascular compression and associated urinary symptoms [15,34].

Limitations

The main limitation of this case report is that the patient’s serum IgG4 level was within the normal range. However, it is important to note that approximately 30% of patients with IgG4-RD may have normal serum IgG4 levels. In such cases, the diagnosis may be based on the clinical presentation and severity of symptoms, characteristic radiological findings, exclusion of other potential causes, and response to treatment.

## Conclusions

In the present case, the diagnosis was established based on the severity of the abdominal pain, after excluding other common causes, together with the characteristic radiological findings. Furthermore, the patient’s symptoms improved following treatment, and a repeat PET-CT scan demonstrated improvement in the previously noted abnormalities, further supporting the diagnosis.

Abdominal pain is one of the most common presenting complaints in the emergency department. Although aortitis is an uncommon cause of abdominal pain, it should be considered in the differential diagnosis of patients presenting with recurrent or unexplained abdominal pain. Early recognition and prompt initiation of appropriate treatment may help prevent complications, including potentially life-threatening ones.
